# Residual coronary malformation after tibial shaft fracture alters the contact status of the meniscus and cartilage in the knee joint: a computational study

**DOI:** 10.3389/fsurg.2024.1325085

**Published:** 2024-09-13

**Authors:** Kai Ding, Wei Liu, Dacheng Sun, Yifan Zhang, Chuan Ren, Xiaodong Cheng, Haicheng Wang, Yanbin Zhu, Xin Xing, Wei Chen

**Affiliations:** ^1^ Department of Orthopaedic Surgery, The Third Hospital of Hebei Medical University, Shijiazhuang, China; ^2^ Key Laboratory of Biomechanics of Hebei Province, Shijiazhuang, Hebei, China; ^3^ Cangzhou People's Hospital, Cangzhou City, Hebei, China; ^4^ NHC Key Laboratory of Intelligent Orthopaedic Equipment (The Third Hospital of Hebei Medical University), Shijiazhuang, Hebei, China

**Keywords:** tibial fractures, varus/valgus deformity, knee joint, mechanical characters, finite element analysis

## Abstract

**Objective:**

The purpose of this study was to evaluate the effect of residual varus/valgus deformity on the mechanical characteristics of the meniscus and cartilage after tibial shaft fracture.

**Methods:**

A finite element model of the lower extremity of a healthy volunteer was constructed from CT and MRI images. The upper and middle tibial fracture models were modified to produce 3°, 5°, and 10° tibial varus/valgus models. For model validation, a patient-specific model with a 10° tibial varus deformity was constructed and simulated under the same boundary conditions.

**Results:**

The contact area and maximum stress of the normal and modified deformity models were similar to those of the reported studies and a patient-specific model. The maximum stress, contact area, and contact force of the medial tibial cartilage in a normal neutral position were 0.64 MPa, 247.52 mm^2^, and 221.77 N, respectively, while those of the lateral tibial cartilage were 0.76 MPa, 196.25 mm^2^, and 146.12 N, respectively. From 10° of valgus to 10° of varus, the contact force, contact area, and maximum stress values of the medial tibial cartilage increased, and those of the lateral tibial cartilage gradually decreased. The maximum stress, contact area, and contact force of the medial tibial cartilage in the normal neutral position were 3.24 MPa, 110.91 mm^2^, and 62.84 N, respectively, while those of the lateral tibial cartilage were 3.45 MPa, 135.83 mm^2^, and 67.62 N, respectively. The maximum stress of the medial tibial subchondral bone in a normal neutral position was 1.47 MPa, while that of the lateral was 0.65 MPa. The variation trend of the medial/lateral meniscus and subchondral bone was consistent with that of the tibial plateau cartilage in terms of maximum stress, contact area, and contact force.

**Conclusion:**

The residual varus/valgus deformity of the tibia has a significant impact on the mechanical loads exerted on the knee joint. This study provides a mechanical basis and references for the clinical evaluation of tibial fracture reduction and osteotomy for tibial deformity.

## Introduction

Tibial fractures represent the most prevalent long bone fracture in clinical practice, accounting for approximately 2% of all cases of fractures (1–3). Tibial shaft fractures are frequently precipitated by high-energy trauma, resulting in extensive tissue damage. A variety of surgical techniques were proposed for the management of tibial fractures, including the use of intramedullary nails, plate fixation, and external fixation (4, 5). However, postoperative complications compromised the surgical outcomes, with reported non-union rates of up to 10% and malunion rates of up to 49% (5–7). Literature review showed that 29%–49% of tibial shaft fractures have an angle of greater than 5° after operation (6, 8). The residual tibial deformities undoubtedly change the normal load transmission of the lower limb, increasing the risk of knee pain and traumatic arthritis (TA) (9, 10). In addition to directly altering the biomechanical properties of the knee resulting in tissue damage, deformity healing can indirectly lead to muscle fatigue, ligament strains, and joint damage by affecting the tension balance in the surrounding muscles and ligaments (11, 12). Weinberg et al. (9) inspected 2,898 cadaveric skeletons and found that the specimens with coronal deformity greater than 5° had an increased incidence of knee arthritis.

A comprehensive grasp of the etiology of TA and an accurate assessment of the biomechanical impact of tibial deformity on the knee are essential prerequisites for the surgical management of tibial shaft fractures and the prevention of traumatic arthritis. However, the current literature is largely limited by epidemiological observational studies, as well as therapeutic studies, and there are few studies on the biomechanics of tibial deformity (13–15). Moreover, defining the deformity parameters largely (10°, 20°, 30°) may be a disadvantage in biomechanical study, as surgical treatment rarely leaves such a large deformity angle. Additionally, while the contact stress and contact area of the knee joint can be obtained in a biomechanical study, the above indicators are challenging to accurately measure the load condition of the knee joint.

Given the above, this study established a finite element model of the lower limb consisting of bone, meniscus, cartilage, and ligaments. The main purposes of this study are to (1) establish a finite element model of tibial varus/valgus deformities and (2) analyze the effect of residual varus or valgus deformity on the contact force, contact area, and stress distribution of the medial and lateral compartments of the knee joint after the middle and upper tibial shaft fracture.

## Materials and methods

### Finite element models

The experiments of this study were performed in accordance with all relevant guidelines and regulations and with the approval of the Institutional Review Board of The Third Hospital of Hebei Medical University. The use of these CT data in this investigation and the experimental protocol of the study were approved by the Third Hospital of Hebei Medical University. Written informed consent was obtained from the volunteers prior to the study commencement.

A healthy male volunteer (age, 30 years; height, 170 cm; body mass, 60 kg) and a male patient (age, 45 years; height, 172 cm; body weight, 60 kg) with tibial varus deformity were scanned by computed tomography (SOMATOM Definition AS Siemens, Germany) with slicing of 0.625 mm from hip joint to the ankle joint. The volunteers’ knee joints were scanned by MRI (MAGNETOM AVanto 1.5 T Siemens, Germany). CT images were used to obtain data on the femur, tibiofibular, and other bone tissues. MRI was used to obtain data on ligaments, menisci, and articular cartilage. The Mimics 21.0 software (Materialise, Leuven, Belgium) was utilized to segment the bone and soft tissue by setting the bone ash threshold to 226, thereby generating a three-dimensional model of the femur through a series of operations, including region growing, masking, segmentation, and smoothing. The three-dimensional model was imported into the Geomagic 2013 software to generate a solid femoral model by denoising and smoothing the surface.

Subsequently, the two-dimensional image data were transformed into a three-dimensional model using Mimics 21.0 software (Materialise, Leuven, Belgium). The apparent density (*ρ*), Young's modulus (*E*), and Poisson's ratio of each element were assigned based on the Hu value in the CT scans according to the following formula (16), which made a distinction between cancellous and cortical bone:

$$\begin{array}{c} \rho (g/cm^{3})=0.000968^{*}HU+0.5\\ \;\mathrm{If}\;\rho <1.2g/cm^{3},E=2014\rho^{2.5},(MPa),v=0.2\\ \;\mathrm{If}\;\rho >1.2g/cm^{3},E=1763\rho^{3.2},(MPa),v=0.32 \end{array}$$

The geometry and surface were built and sampled by the Geomagic software. The normal lower extremity model was imported into UG NX 9.0 (Siemens Product Life Cycle Management Software, Inc., USA). The mechanical axis of the lower extremities was determined on the three-dimensional model according to the method proposed by Moreland et al. and Whiteside et al. (17, 18). The proximal and middle tibial fracture models were created and rotated 3°, 5°, and 10° medially or laterally, centered on the mechanical axis. There were six models of varus and valgus deformities, both each at 3°, 5°, or 10°. The normal lower extremity model was imported into Hypermesh 14.0 (Altair Engineering, Troy, MI, USA). The cartilage, meniscus, and bone models were constructed with C3D4 elements (Table 1). The models were exported to the finite analysis software Abaqus 6.14 (Simulia Corp., Providence, RI, USA).

**Table 1:** Amounts of nodes and elements of five components.

Components	Nodes	Elements
Femur	6,759	32,898
Femoral articular cartilage	1,640	25,057
Meniscus	2,883	11,957
Tibial articular cartilage	5,854	16,858
Tibia and fibula	23,610	94,440

### Material properties and boundary conditions

We assumed that all models were homogeneous, isotropic, and linearly elastic. According to early scholars (19–21), articular cartilage, ligaments, and meniscus were assigned a Young's modulus (*E*) of 5 MPa, 215.3 MPa, and 59 MPa, respectively, and the Poisson's ratio was 0.46, 0.46, and 0.49, respectively. The anterior and posterior horns of the meniscus were connected with the tibial plateau by ten spring elements, and the spring stiffness was set at 200 N/mm (22).

**Figure 1:**
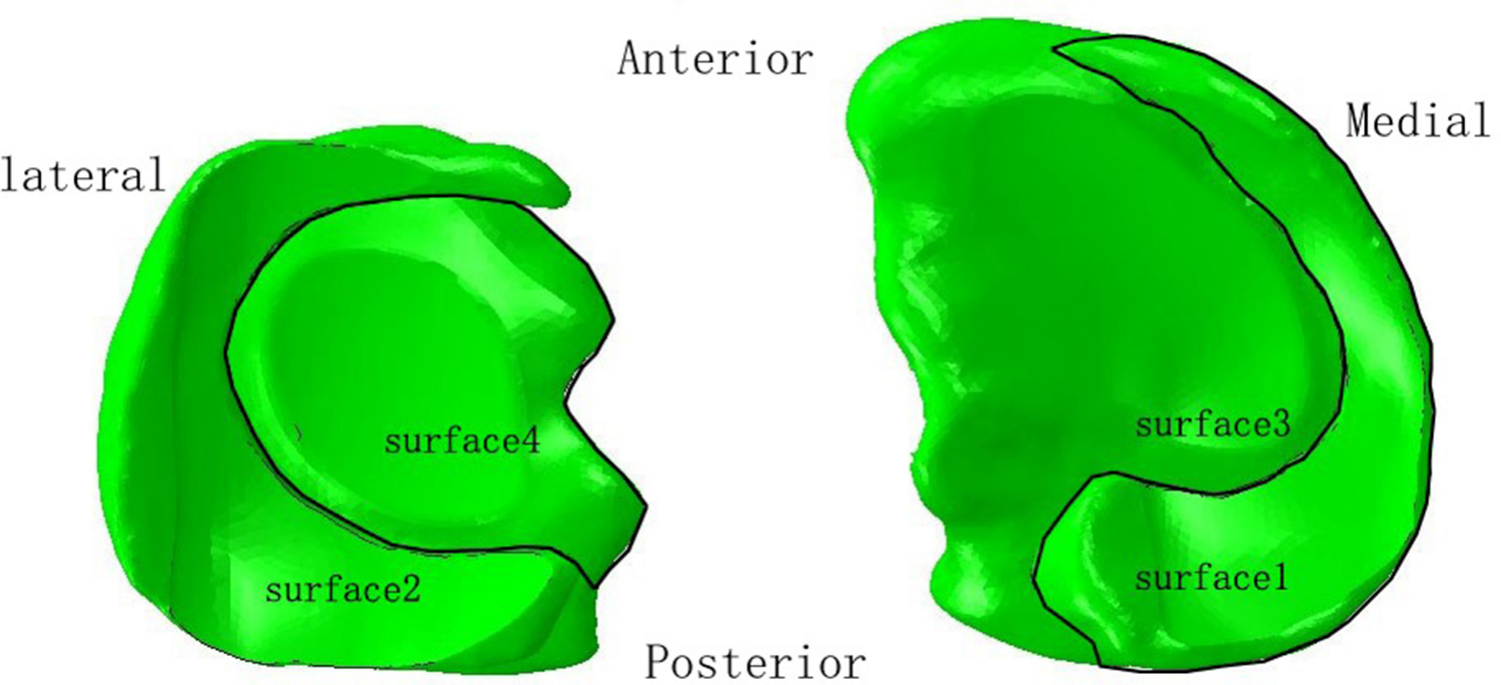
Diagram of four contact relationships.

In the lower extremity model, four contact relationships were established. We defined the contact area between the medial femoral condylar cartilage and the medial meniscus, between the lateral femoral condylar cartilage and the lateral meniscus, between the medial femoral condylar cartilage and the medial tibial plateau cartilage, and between the lateral femoral condylar cartilage and the lateral tibial plateau cartilage as surface1, surface2, surface3 and surface4, respectively (Figure 1). The contact relationship was set as “hard contact between surface and surface” and “frictionless finite sliding,” which was used to simulate the finite sliding state of the knee joint. Ligaments and springs were bound to the corresponding regions. The tied relationships were used for malunited sites.

In this experiment, the lower extremity was loaded with a downward vertical force of 600 N (one-leg standing load force of 100% of the body weight; the gravitational acceleration, 10 m/s^2^) (23, 24). The distal tibiofibula was fixed in all degrees of freedom based on deformity extent (3°, 5°, and 10° at varus and valgus, respectively).

### Validation of finite element models

**Figure 2:**
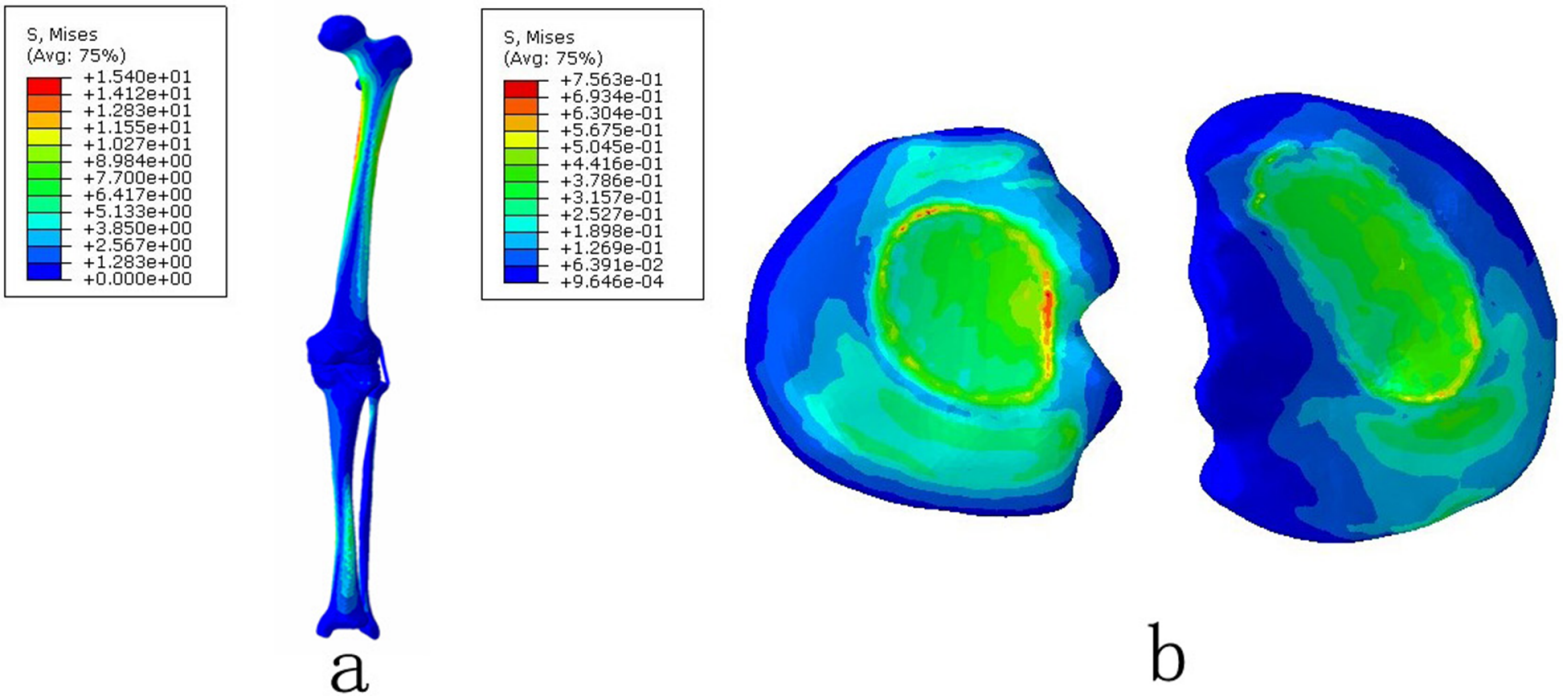
The von Mises stress distribution on the lower extremity under the normal natural position: **(a)** the complete lower extremity and **(b)** the tibial plateau cartilage.

**Figure 3:**
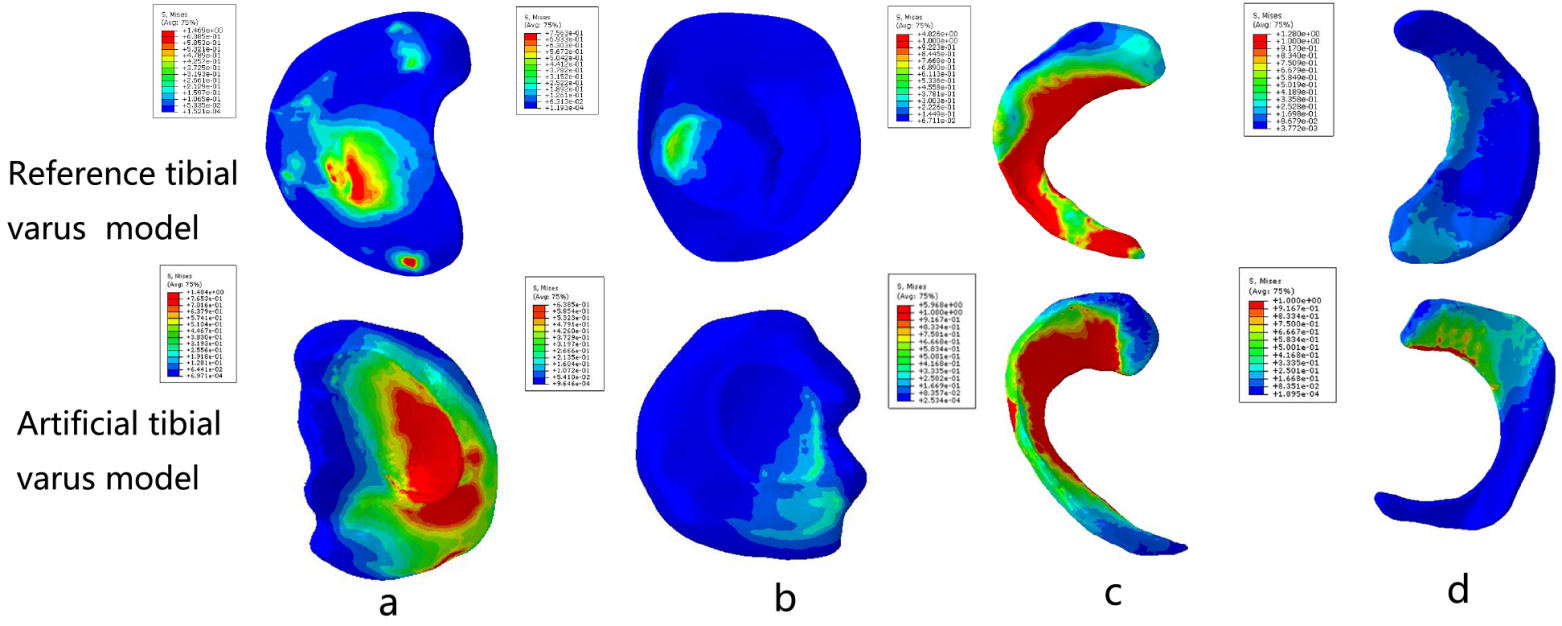
Comparison between the real varus tibial model and the artificial varus tibial model: **(a)** the medial tibial plateau cartilage; **(b)** the lateral tibial plateau cartilage; **(c)** the medial meniscus; and **(d)** the lateral meniscus.

The grid convergence calculations were tested by different sizes. The convergence criterion used was a change of <5%. To make a comparison with previous studies (25, 26), we applied a vertical load of 1,000 N and 2,400 N to the femoral head to obtain the contact area and maximum contact stress of the knee joint, respectively (Table 2). Furthermore, a patient-specific model with a 10° angle tibial varus was also created to validate the artificial deformed model. In this study, the maximum stress values and contact area of tibial cartilage and meniscus in neutral normal position were compared with the reported results (25, 26) and the patient-specific model to evaluate the effectiveness of modeling. The results of the comparison ensured that our modeling method was convincing and reliable (Figures 2, 3) (Table 2).

**Table 2:** Comparisons of contact area and von Mises stress of this study and previous studies.

Study		Contact area (cm^2^)		Maximum stress (MPa)	
	Applied load (*N*)	Medial compartment	Lateral compartment	Medial compartment	Lateral compartment
Present study	2,400	5.46	4.78	6.35	7.64
McKellop et al. (17)	2,400	7.9	5.9	4.1	4.6
Present study	1,000	4.01	3.75	5.37	6.65
Morimoto et al. (27)	1,000	5.95 ± 1.54	4.44 ± 1.07	4.76 ± 1.2	5.24 ± 1.0

## Results

### Stress distribution of the medial and lateral compartments of the knee joint under the tibial varus and valgus deformities

**Figure 4:**
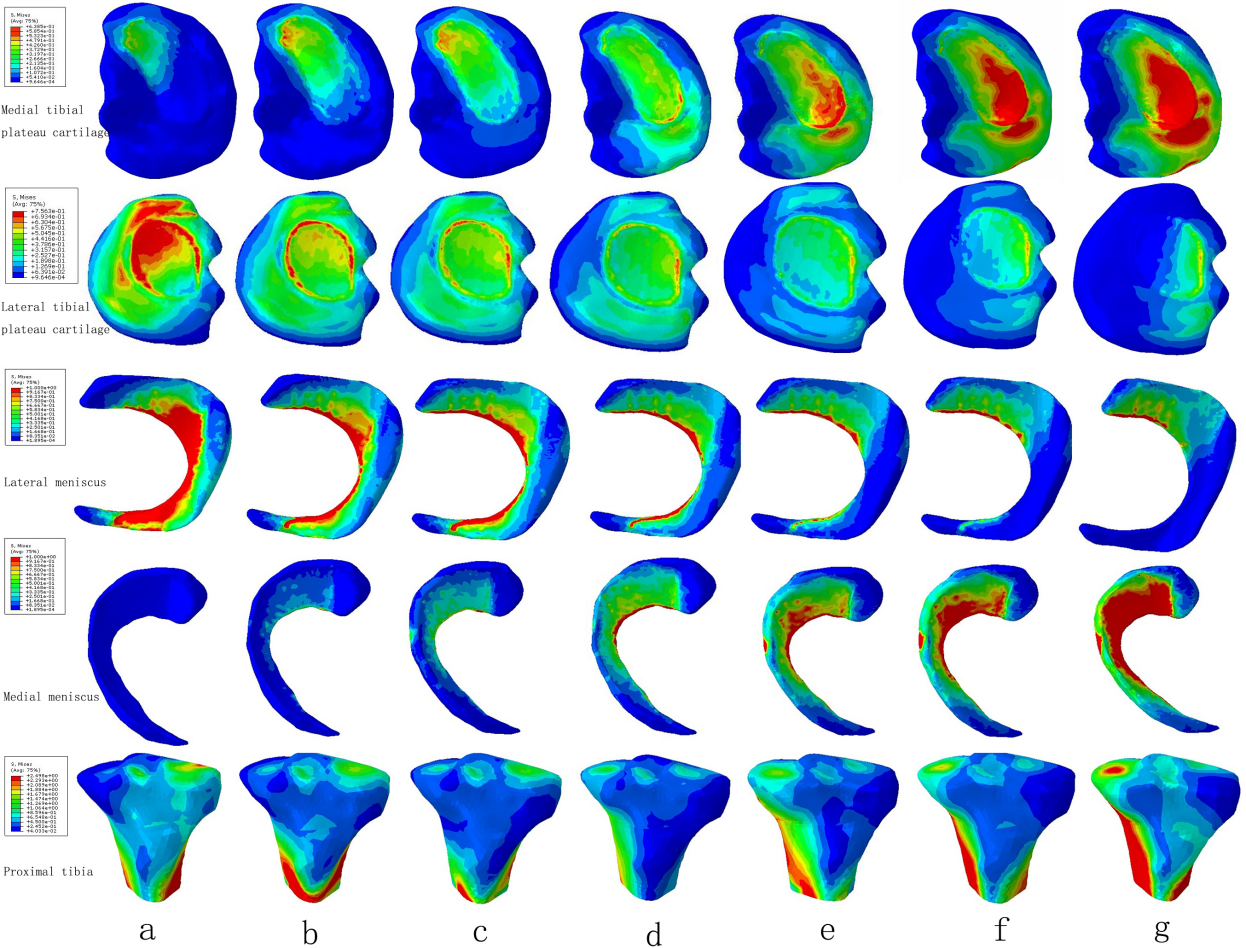
The von Mises stress distribution on the tibial plateau cartilage, meniscus, and proximal tibia under the condition of varus and valgus deformities of the tibia: **(a)** valgus 10°; **(b)** valgus 5°; **(c)** valgus 3°; **(d)** neutral position 0°; **(e)** varus 3°; **(f)** varus 5°; and **(g)** varus 10°.

**Figure 5:**
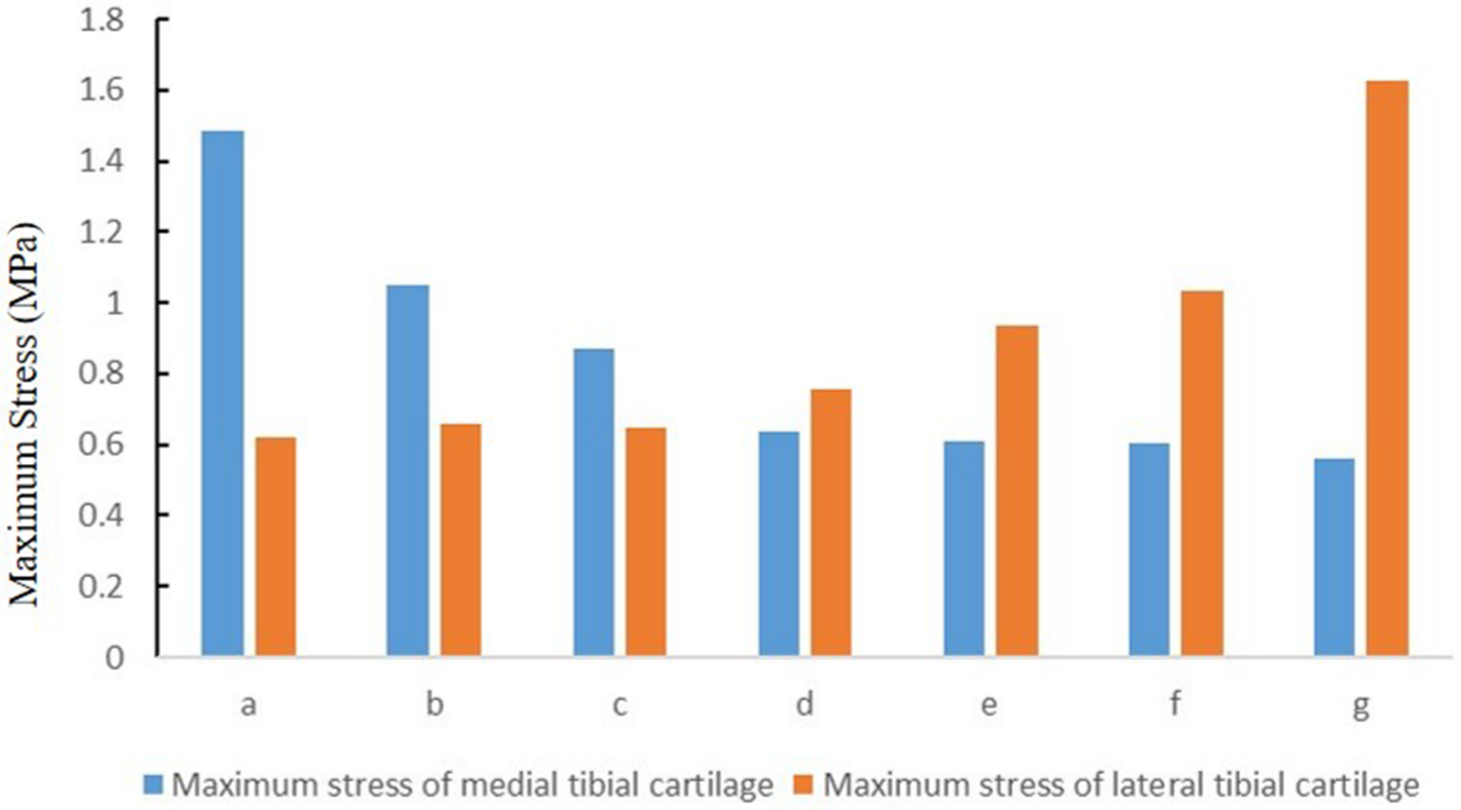
The stress distribution of cartilage under the condition of varus and valgus deformities of the tibia: **(a)** valgus 10°; **(b)** valgus 5°; **(c)** valgus 3°; **(d)** neutral position 0°; **(e)** varus 3°; **(f)** varus 5°; and **(g)** varus 10° (MPa).

The maximum stress of medial tibial cartilage, tibial subchondral bone, and meniscus in a normal neutral position were 0.64 MPa, 0.65 MPa, and 3.24 MPa, while those of the lateral were 0.76 MPa, 1.47 MPa, and 3.45 MPa, respectively. From the neutral position to 10°of varus, the maximum stress of medial tibial cartilage, tibial subchondral bone, and meniscus increased to 1.48 MPa, 0.65 MPa, and 6.73 MPa, and those of the lateral gradually decreased to 0.62 MPa, 0.65 MPa, and 1.84 MPa, respectively. From the normal neutral position to 10°of valgus, the maximum stress of medial tibial cartilage, tibial subchondral bone, and meniscus gradually decreased to 0.55 MPa, 0.87 MPa, and 1.65 MPa, while those of the lateral increased to 1.63 MPa, 2.75 MPa, and 6.01 MPa, respectively (Figures 4 and 5).

### The contact area of the medial and lateral compartments of the knee joint under the tibial varus and valgus deformities

**Figure 6:**
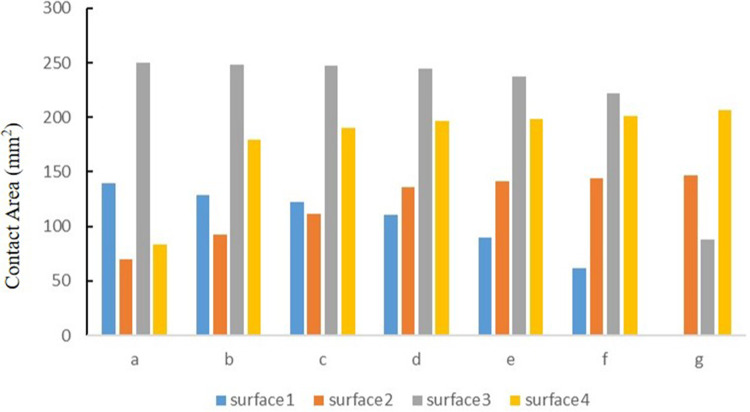
The contact area of four contact relationships under the condition of varus and valgus deformities of the tibia: **(a)** valgus 10°; **(b)** valgus 5°; **(c)** valgus 3°; **(d)** neutral position 0°; **(e)** varus 3°; **(f)** varus 5°; and **(g)** varus 10° (mm^2^).

The contact area of the medial tibial cartilage and meniscus in a normal neutral position was 247.52 mm^2^ and 110.91 mm^2^, while that of the lateral was 196.25 mm^2^ and 135.83 mm^2^, respectively. From the neutral position to 10°of varus, the contact area of medial tibial cartilage and meniscus increased to 250.12 mm^2^ and 139.46 mm^2^, and that of the lateral gradually decreased to 83.68 mm^2^ and 69.38 mm^2^, respectively. From the normal neutral position to 10°of valgus, the contact area of medial tibial cartilage and meniscus gradually decreased to 87.94 mm^2^ and 0 mm^2^, while that of the lateral increased to 205.51 mm^2^ and 146.87 mm^2^, respectively (Figure 6).

### The contact force of the medial and lateral compartments of the knee joint under the tibial varus and valgus deformities

**Figure 7:**
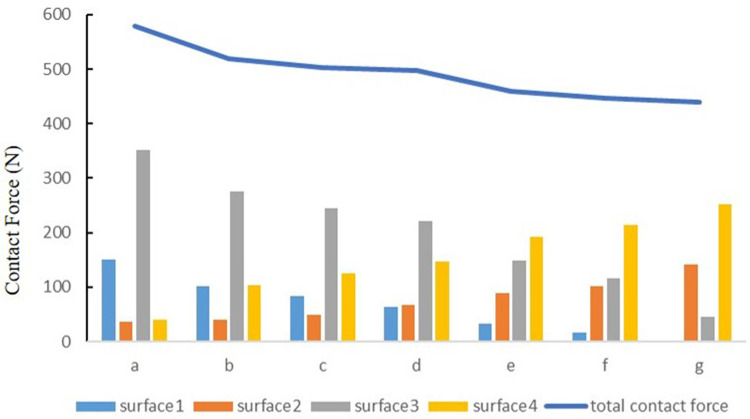
The contact force of four contact relationships under the condition of varus and valgus deformities of the tibia: **(a)** valgus 10°; **(b)** valgus 5°; **(c)** valgus 3°; **(d)** neutral position 0°; **(e)** varus 3°; **(f)** varus 5°; and **(g)** varus 10° (***N***).

The contact force of the medial tibial cartilage and meniscus in a normal neutral position was 221.77 N and 62.84 N, while that of the lateral was 146.12 N and 67.62 N, respectively. From the neutral position to 10°of varus, the contact force of medial tibial cartilage and meniscus increased to 351.55 N and 150.08 N, and that of the lateral gradually decreased to 40.71 N and 35.77 N, respectively. From the normal neutral position to 10°of valgus, the contact force of medial tibial cartilage and meniscus gradually decreased to 45.56 N and 0 N, while that of the lateral increased to 252.76 N and 140.91 N, respectively (Figure 7).

## Discussion

The varus/valgus deformity is a common complication after a tibial fracture. The tibial deformity is closely related to the occurrence and development of knee TA. Our findings demonstrate that the varus/valgus deformity of the tibia significantly alters the contact force, contact area, and stress of the knee's medial and lateral compartments, providing the biomechanical evidence to support the potential association between tibial deformity and knee TA.

The results demonstrate that the medial compartment exhibits a larger size than the lateral compartment with regard to both contact force and contact area. As the degree of varus deformity increases, the contact force, contact area, and stress peak value of the medial compartment gradually increase, while those of the lateral compartment gradually decrease. It is the opposite of valgus deformities. A tibial deformity alters the balance of the medial–lateral compartment of the normal neutral position by influencing the mechanical axis of the lower extremity, which may shift inward or outward. Varus and valgus deformities of the lower limb have increased the rate of knee arthritis, especially in obesity and overweight groups (9, 27, 28). Compared to previous studies (13, 15), the contact force of the meniscus and tibial cartilage is additionally recorded to reflect the load of the knee joint in the current study. However, the total contact force of normal and malunion models differs and is lower than 600 N. This is mainly caused by shorter tibial moments of the deformity and traction of ligaments around the knee joint. Moreover, the contact force of 10° of varus in the medial compartment is 1.76 times the normal values, and the contact force of 10° of valgus in the lateral compartment is 1.84 times the normal values. We suggest that tibial varus and valgus deformities have a symmetric effect on the knee joint in a finite element model of the lower limb. In this study, a finite element model of the lower limb was reconstructed, and the center of the femoral head to the center of the ankle joint was set as the lower limb of the mechanical axis, which avoided the deviation of the mechanical axis generated by a portion of the lower limb.

Tibial varus/valgus deformities alter the mechanical characteristics of the knee joint, thereby increasing the likelihood of knee arthritis. Firstly, for the injury mechanism of meniscus and tibial cartilage, tibial varus and valgus deformities caused an increase in the load and highly stressed zone of tibial cartilage and meniscus. According to the findings of a population baseline survey, the incidence of medial cartilage injury in individuals with varus malalignment is 3.59 times that observed in the normal population (29). Felson et al. (30) reported that valgus malalignment has a strong relation with lateral meniscal and cartilage damage. Furthermore, some studies have suggested that knee varus/valgus malalignment is an important risk factor for anterior cruciate ligament (ACL) injury (31, 32). Finally, subchondral osteosclerosis represents a significant clinical manifestation of knee arthritis, resulting from bone remodeling under conditions of excessive abnormal load. Bobinac et al. (33) suggested that subchondral bone morphological changes are even faster than cartilage changes in patients with knee arthritis. Tibial deformity has been demonstrated to significantly alter the stress distribution of the knee subchondral bone, which will ultimately lead to sclerosis of the knee subchondral bone.

The knee joint is composed of a variety of tissues, and damage to any tissue can affect the function and structure of other tissues as well. Damage to the ACL can result in tears to the meniscus, chronic knee instability, cartilage damage, and knee arthritis, due to alterations in the stability and alignment of the knee joint (31, 34, 35). Injuries to the meniscus can also lead to cartilage destruction and knee arthritis by changing the stability of the knee joint (36, 37). Additionally, sclerosis of the subchondral bone can result in a reduction of the cushioning force, leading to stress concentration in the cartilage and subsequent damage to the cartilage (38). The meniscus, cartilage, cruciate ligament, and subchondral bone of the knee joint are closely related to each other in tissue structure and function (39). Tibial varus and valgus deformities could cause damage to the normal structure of the knee joint, and various structures also interact, together leading to an increased incidence of knee arthritis for a long time. Therefore, early osteotomy can avoid the occurrence and progression of knee TA. To accurately construct soft tissue models, such as knee ligaments, we employed a knee brace to maintain the knee in a state of extension, thereby limiting the range of motion and reducing the discrepancy between CT and MRI images. Furthermore, this study also drew upon data reported in the literature and human anatomy to determine the initial and final points of the ligaments (40–42). The finite element model of the ligament was then constructed with precision through the aforementioned methods.

In addition to knee replacement, osteotomy is an important treatment to correct tibial varus and valgus deformities especially in the young population. However, correction goals for osteotomy are currently controversial. Coventry et al. (43) suggested that the intended tibiofemoral correction angles for varus knee was overcorrection to a lower limb anatomical axis of 8°–10° of valgus angulation. Hernigou et al. (44) recommended that a postoperative mechanical axis of 3°–6° of valgus angulation should be maintained. Jung et al. (45) demonstrated that cartilage regeneration in the medial compartment of knees with standard correction is superior to that observed with overcorrection and undercorrection, with respect to both cartilage regeneration and clinical outcomes. Nevertheless, some studies have indicated that there is no significant distinction in the impact of overcorrection and standard osteotomy on the knee joint (46, 47). Naudie et al. (47) posited that the intended mechanical axis of the lower limb should be maintained within the normal range. This study aimed to investigate the relationship between the magnitude of the deformity and the load in the medial and lateral compartments of the knee joint. This will enable the load in the medial and lateral compartments of the knee joint to be distributed quantitatively, thus providing an accurate reference for clinical osteotomy. Finally, the degree of osteotomy has not been quantified in the long-term prognosis of osteotomy. Future studies should still focus on the relationship between the overall survival time of osteotomy and loads.

This study has shown that tibial varus and valgus deformity can significantly affect the biomechanical properties of the knee joint. Therefore, our findings can provide clinical evidence on two aspects of tibial fracture management. Firstly, the reduction of the tibial shaft fracture should be kept in a clean alignment, and the fracture reduction should be more finely adjusted to keep the internal and external deformity angle less than 5°. Secondly, in view of the above evidence, the tibial internal and external deformity should be corrected aggressively for the deformity angle greater than 5° to prevent long-term complications such as arthritis.

Our study is not without limitations. First, this study simulates the human standing on one foot, ignoring the lower limb muscles, which may be different from the real load condition of the human body. Second, all models were assigned isotropy and homogeneity to simplify the model. However, the finite element model of the lower limb was validated with reported research, and the difference is within acceptable limits. Finally, the finite element model of the lower limb in this study does not include the foot, which may be different from the real lower limb.

In conclusion, tibial varus/valgus deformity has a significant influence on the contact force, contact area, and stress of the medial and lateral compartments of the knee joint. This study provides a mechanical basis and reference for clinical evaluation of tibial fracture reduction and osteotomy for tibial deformity.

## Data Availability

The original contributions presented in the study are included in the article/Supplementary Material; further inquiries can be directed to the corresponding authors.
